# Association Between Serum High Sensitivity C-Reactive Protein Levels and Low Muscle Strength Among Korean Adults

**DOI:** 10.3390/nu17162698

**Published:** 2025-08-20

**Authors:** Bo-Hyun Choi, Sunhye Shin

**Affiliations:** 1 Department of Pharmacology, School of Medicine, Daegu Catholic University, Daegu 42472, Republic of Korea; bchoi@cu.ac.kr; 2 Department of Food and Nutrition, Seoul Women’s University, Seoul 01797, Republic of Korea

**Keywords:** high sensitivity C-reactive protein, muscle strength, handgrip strength, sarcopenia, nutrition survey, adults

## Abstract

Purpose: Chronic low-grade inflammation is increasingly recognized as a contributor to age-related muscle loss and functional decline, yet its association with muscle strength in Asian populations remains underexplored. Therefore, this study aimed to investigate the relationship between high-sensitivity C-reactive protein (hsCRP) and low muscle strength in Korean adults. Materials and Methods: Data were obtained from 14,354 participants aged ≥ 19 years in the 7th Korea National Health and Nutrition Examination Survey (KNHANES VII, 2016–2018). Low muscle strength was defined as handgrip strength < 28 kg for men and <18 kg for women, and serum hsCRP levels were categorized as normal (<1.0 mg/L), elevated (1.0–3.0 mg/L), and high (≥3.0 mg/L). Multivariable logistic regression was used to assess the association between serum hsCRP level and low muscle strength with adjustment for possible confounders. Results: Among Korean adults, 27.7% had elevated or high hsCRP level, and low muscle strength was prevalent in older adults ≥ 65 years (men 22.7%, women 34.1%). Elevated hsCRP was associated with increased odds of low muscle strength in middle-aged women 40–64 years (odds ratio [OR], 1.47; 95% confidence interval [CI], 1.04−2.09) and in older women ≥ 65 years (OR, 1.34; 95% CI, 1.04−1.74). High hsCRP was associated with higher risk in older men (OR, 1.71; 95% CI, 1.06–2.75) and older women (OR, 1.66; 95% CI, 1.14–2.42). Conclusions: Higher hsCRP levels were independently associated with low muscle strength in middle-aged women and older adults. Downregulating inflammation through nutritional strategies could help prevent muscle decline with aging.

## 1. Introduction

Sarcopenia is not only associated with aging but also with increased risks of frailty, falls, functional impairment, disability, and all-cause mortality, particularly in older adults [1]. Traditionally, sarcopenia has been viewed primarily as a consequence of chronological aging and physical inactivity; however, recent advances in geroscience have shifted the paradigm, suggesting that biological processes such as chronic inflammation may play an equally critical role in its development and progression [2,3].

Low muscle strength is a core component of sarcopenia, a progressive and multifactorial condition characterized by the deterioration of skeletal muscle mass, strength, and function [4]. Muscle strength can directly measure muscle function, and impaired muscle function is considered as the most reliable predictor of health outcomes, such as hospital stay and death [5]. Therefore, low muscle strength is recognized as probable or possible sarcopenia in multiple consensus definitions [2,6].

Among the various biological contributors to muscle degradation, systemic inflammation has emerged as a central factor in age-related muscle decline [7,8]. Pro-inflammatory cytokines, such as interleukin-6 (IL-6) and tumor necrosis factor-alpha (TNF-α), are known to activate proteolytic pathways, suppress muscle protein synthesis, and interfere with satellite cell-mediated regeneration. This low-grade, chronic inflammatory state—often referred to as “inflammaging”—has been linked to anabolic resistance, mitochondrial dysfunction, and neuromuscular degradation, all of which accelerate sarcopenic trajectories in older adults [7,8]. As well as infectious and allergic agents, nutritional factors, such as the quantity of overall dietary intake and specific nutrients, significantly contribute to chronic inflammation [9]. Therefore, nutritional strategies are required to decrease the risk of muscle-related disorders.

C-reactive protein (CRP), particularly measured using high-sensitivity CRP assays (hsCRP), is one of the most accessible and widely used clinical biomarkers of systemic inflammation [10]. HsCRP reflects subtle increases in inflammatory burden and has been consistently associated with several adverse health outcomes, including cardiovascular disease, metabolic syndrome, insulin resistance, and physical disability [11,12,13]. It is also considered a reliable surrogate marker for IL-6 and TNF-α activity, providing a practical tool to assess inflammatory status in large population studies [10].

Although previous studies have reported associations between CRP or hsCRP levels and low muscle strength or reduced physical performance, the findings are inconsistent [13,14]. It seems that the variability in results is due to differences in study design, participant characteristics, methods of strength measurement, and definitions of inflammation thresholds. In addition, the associations appear to vary by sex and age, suggesting the presence of biologically meaningful interactions among hormonal status, body composition, and inflammation-mediated muscle degradation [13,14].

In the Korean context, the growing proportion of older adults has heightened awareness of sarcopenia as a public health issue [15]. However, despite the increasing burden of age-related functional decline, there remains a lack of nationally representative data exploring the relationship between systemic inflammation and muscle strength in Korean adults. Existing studies have either focused solely on the elderly or lacked sex- and age-stratified analysis, limiting the generalizability and clinical applicability of their findings. Furthermore, no consensus has yet been reached on whether hsCRP could serve as an early, modifiable marker for muscle dysfunction across different age groups.

Therefore, we hypothesized that serum hsCRP levels are associated with low muscle strength in Korean adults and analyzed blood profiles and handgrip strength from the 7th Korea National Health and Nutrition Examination Survey (KNHANES VII). By stratifying participants by age and sex, we aimed to identify specific population groups who may be at greater risk of inflammation-associated muscle weakness. Understanding these associations in a nationally representative Korean cohort will not only contribute to the growing body of literature on sarcopenia and inflammation but also inform preventive strategies and early interventions that could help mitigate functional decline in aging populations.

## 2. Methods

### 2.1. Data Source and Subjects

This study utilized data from the 7th Korea National Health and Nutrition Examination Survey (KNHANES VII), a nationwide, cross-sectional program that provides representative health statistics for the Korean population. The survey comprises three main components: a health interview, a health examination, and a nutrition assessment [16]. Data collection for KNHANES VII received approval from the Institutional Review Board (IRB) of the Korea Centers for Disease Control and Prevention (IRB No. 2018-01-03-P-A) [17]. All participants provided informed consent. For the present analysis, ethical review and approval were waived by the IRB of Seoul Women’s University for this study (IRB No. SWU IRB-2023A-02, approval date: 12 April 2023).

Among the 24,269 individuals originally enrolled in the KNHANES VII, children ≤ 18 years (*n* = 4880), and individuals missing dietary intake data (*n* = 2535), handgrip strength data (*n* = 1455), and hsCRP value (*n* = 1045) were serially excluded. Consequently, 14,354 participants were analyzed after categorization by age and sex for potential biological differences as follows: men 19–39 years (*n* = 1720), women 19–39 years (*n* = 2176), men 40–64 years (*n* = 2859), women 40–64 years (*n* = 3891), men ≥ 65 years (*n* = 1651), and women ≥65 years (*n* = 2057).

### 2.2. General Characteristics and Dietary Intake

The information on demographic details, socioeconomic status, personal behaviors, and medical conditions were obtained through structured health interviews. Household income was divided into four quartiles: low, middle-low, middle-high, and high. Participants who reported drinking alcohol at least once per month during the past year were considered current drinkers. Current smokers were defined as those who had smoked over 100 cigarettes in their lifetime and continued smoking at the time of the survey. Regular aerobic activity was defined as engaging in at least 150 min of moderate-intensity exercise, 75 min of vigorous-intensity exercise, or a combination of both each week, with 1 min of vigorous physical activity counted as equivalent to 2 min of moderate activity. Chronic medical conditions included type 2 diabetes, cancers, stroke, cardiovascular diseases, or osteoarthritis, as these illnesses are known to affect protein consumption and/or muscle loss [18].

Dietary intake data were collected by trained dietitians using a single 24 h dietary recall interview. Daily intakes of total energy, food items, and nutrients were estimated based on the KNHANES recipe database and the Korean Rural Development Administration’s food composition tables [19].

### 2.3. Health Examinations

Body weight and height were measured, and blood samples were collected for biochemical analysis. Body mass index (BMI) was determined by dividing body weight (kg) by the square of height (m^2^). Based on the statement of the Centers for Disease Control and the American Heart Association, serum hsCRP level < 1.0, 1.0−3.0, and ≥3.0 mg/L were categorized as normal, elevated, and high hsCRP level, respectively [20].

After participants shake both hands 3 times and gently clench and unclench all fingers 3 times, handgrip strength was measured when participants stood upright and with extended elbow using the Digital Grip Strength Dynamometer (T.K.K 5401, Takei Scientific Instruments, Tokyo, Japan). To consistently measure the grip strength of the participants, the investigators demonstrated the procedure after the explanation. The value was expressed in kilograms, and this study used the maximum value of three trials with the dominant hand [21]. In line with the Asian Working Group for Sarcopenia 2019 consensus and Korean Working Group on Sarcopenia Guideline, handgrip strength < 28 kg for men and <18 kg for women were classified as low handgrip strength [6,22].

### 2.4. Statistical Analysis

Participant characteristics according to hsCRP quartiles were summarized as means ± standard errors (SE) for continuous variables and as frequencies with percentages for categorical variables. Differences between groups were evaluated using Student’s *t*-test for continuous variables and Rao-Scott chi-square tests for categorical variables.

The association between hsCRP level and the likelihood of low muscle strength was examined using multivariate logistic regression, with the lowest hsCRP quartile serving as the reference. Four models were constructed: the unadjusted model provided crude odds ratios (ORs) and 95% confidence intervals (CIs); Model 1 adjusted for age, BMI, and total energy intake; Model 2 was further accounted for household income, alcohol use, smoking habits, regular resistance exercise, and chronic medical conditions; and Model 3 additionally included protein-derived energy intake.

All statistical analyses were conducted using SPSS software (Version 26, IBM, Armonk, NY, USA) based on the survey procedure [16]. Statistical significance was set at a two-sided *p*-value < 0.05.

## 3. Results

Approximately 27.7% of Korean adults had the hsCRP level above the normal value (≥1.0 mg/L). By age and sex, 28.2% among younger men 19–39 years, 23.0% of younger women 19–39 years, 30.3% of middle-aged men 40–64 years, 23.9% of middle-aged women 40–64 years, 34.9% of older men ≥ 65 years, and 31.9% of older women ≥ 65 years had elevated or high hsCRP level.

The prevalence of low muscle strength among Korean adults was 8.5%. Older women ≥65 years had the highest prevalence of low muscle strength (34.1%), followed by older men ≥65 years (22.7%), middle-aged women 40–64 years (6.8%), younger women 19–39 years (6.6%), younger men 19–39 years (2.4%), and middle-aged men 40–64 years (2.2%).

Table 1 shows the distribution of general characteristics of younger adults 19–39 years according to serum hsCRP level. Younger adults with normal hsCRP level tended to do more resistance exercise and have lower BMI. Younger men with normal hsCRP level were tended to be younger, and younger women with normal hsCRP level were more likely to have more household income and be current alcohol consumer.

Table 2 shows the distribution of general characteristics of middle-aged adults 40–64 years according to serum hsCRP level. Middle-aged adults with normal hsCRP level were more likely to have higher household income and lower BMI. Middle-aged men with normal hsCRP level were less likely to be current smoker and tended to have higher maximal handgrip strength. Middle-aged women with normal hsCRP level tended to be younger, were more likely to have more household income, and do more resistance exercise.

Table 3 shows the distribution of general characteristics of older adults ≥ 65 years according to serum hsCRP level. Older adults with normal hsCRP level tended to be younger, and have higher maximal handgrip strength. Older men with high hsCRP level were less likely to do resistance exercise, and older women with high hsCRP level were more likely to be current alcohol consumer.

As a continuous variable, hsCRP level was associated with maximal handgrip strength (Table 4). Although no correlation was shown in younger women and older men, hsCRP level was negatively correlated with handgrip strength after adjusting possible confounders in younger men 19–39 years (β = −0.350; SE = 0.081; *p*-value < 0.001), middle-aged men (β = −0.136; SE = 0.066; *p*-value = 0.041) and women 40–64 years (β = −0.096; SE = 0.048; *p*-value = 0.048), and older women ≥65 years (β = −0.160; SE = 0.051; *p*-value = 0.002).

Associations between hsCRP and low muscle strength was shown among middle-aged women 40–64 years and older adults ≥ 65 years (Table 5). After adjusting possible confounding variables, middle-aged women with elevated hsCRP level (1.0−3.0 mg/L) had 47% higher risk (OR, 1.47; 95% CI, 1.04−2.09) of low muscle strength, and older men with high hsCRP level (≥3.0 mg/L) had 71% higher risk (OR, 1.71; 95% CI, 1.06−2.75) compared to middle-aged women and older men with normal hsCRP level, respectively. Older women with elevated and high hsCRP level had 34% (OR, 1.34; 95% CI, 1.04−1.74) and 66% higher risk (OR, 1.66; 95% CI, 1.14−2.42) of low muscle strength than those with normal hsCRP level, respectively.

## 4. Discussion

In this large, nationally representative study of Korean adults, we observed that elevated or high levels of hsCRP were significantly associated with increased odds of low muscle strength, especially in middle-aged women and older adults. These findings persisted even after adjusting for a comprehensive set of potential confounders, including age, BMI, energy intake, household income, alcohol consumption, smoking, resistance exercise, medical condition, and energy from protein. The strength and consistency of these associations support the growing recognition of chronic low-grade systemic inflammation as a critical contributor to muscle dysfunction and sarcopenia, a condition traditionally attributed to aging and disuse [3,23].

The biological plausibility of our findings is reinforced by a robust body of evidence linking inflammatory signaling to the degradation of skeletal muscle. Pro-inflammatory cytokines such as IL-6 and TNF-α have been shown to activate proteolytic pathways, impair mitochondrial function, reduce anabolic signaling, and suppress the regenerative capacity of satellite cells—all of which contribute to the loss of muscle mass and strength [24]. HsCRP, while a nonspecific marker, is a clinically accessible indicator of these underlying processes because IL-6 signaling additionally enhanced by TNF-α increases CRP transcription rate [10]. In our study, participants with hsCRP levels above 1.0 mg/L—classified as elevated or high—demonstrated significantly greater risk of low muscle strength, reinforcing the potential utility of hsCRP as a surrogate marker for inflammation-related musculoskeletal decline [25].

Interestingly, the association was more pronounced in women, particularly in the postmenopausal age group. This observation is consistent with prior research suggesting that estrogen exerts protective effects on muscle via both anti-inflammatory and anabolic pathways [14]. The decline in estrogen after menopause may heighten vulnerability to inflammatory insults, thereby accelerating muscle degradation in women. Additionally, older adults are more likely to experience age-related immune dysregulation, often described as “inflammaging,” which is characterized by increased circulating inflammatory markers even in the absence of acute illness [26]. This chronic, smoldering inflammation may explain the stronger associations observed in the older age strata.

By contrast, we did not observe significant associations between hsCRP and low muscle strength in younger adults aged 19–39 years. This may reflect their overall lower inflammatory burden, greater physiological resilience, and higher muscle regenerative capacity. It is also plausible that younger individuals have not experienced long enough exposure to systemic inflammation to manifest clinically relevant impairments in muscle strength. These findings highlight the importance of identifying at-risk individuals in midlife, where interventions targeting inflammation could prevent or delay the onset of muscle decline and functional impairment in later life [27].

HsCRP has also been shown to be associated with the risk of sarcopenia in Western populations. Among the general population in the United States, adults in the highest quartile of CRP level had 2.74 higher risk of sarcopenia than those in the lowest quartile [28], and increased hsCRP/high-density lipoprotein cholesterol (HDL-C) ratio, a marker integrating inflammation and lipid metabolism, was associated with the higher sarcopenia risk [29].

Our results have several important clinical and public health implications. First, they suggest that hsCRP, an inexpensive and routinely available biomarker in clinical practice, could be repurposed beyond its traditional use in cardiovascular risk stratification to identify individuals at risk for sarcopenia [12]. Early identification could guide targeted interventions such as resistance exercise programs, nutritional optimization, and anti-inflammatory lifestyle modifications. Second, the data support the hypothesis that systemic inflammation is not merely an association but may be a mechanistic contributor to muscle loss. This opens the door to novel therapeutic strategies that include pharmacological modulation of inflammation, in addition to established behavioral approaches [30].

From a public health perspective, these findings are particularly relevant in countries like Korea, where the aging population is rapidly increasing, and where sarcopenia is emerging as a major contributor to healthcare burden due to its impact on mobility, independence, and quality of life. The identification of hsCRP as a potential biomarker for muscle weakness could inform screening strategies in national health check-up programs and promote earlier dietary and lifestyle interventions, particularly among women and older adults. Since dietary intake of anti-inflammatory nutrients, including *n*-3 fatty acids [31,32], vitamin C [33,34], and dietary fibers [35,36], was shown to be associated with reduced CRP level and reduced risk of low muscle strength, dietary intervention targeting hsCRP may be effective against possible sarcopenia.

Despite these strengths, several limitations must be acknowledged. The cross-sectional design of the study precludes any causal inference. While we observed associations between elevated hsCRP levels and low muscle strength, we cannot determine the directionality of the relationship. It is possible, for example, that low muscle mass or function itself contributes to systemic inflammation, rather than the reverse. Longitudinal studies are needed to disentangle these relationships. Additionally, handgrip strength is a surrogate marker and assesses isometric contraction; therefore, other tests to objectively evaluate muscle function, such as surface electromyography and musculoskeletal ultrasound, or determine dynamic strength are required to clarify the association. Moreover, although hsCRP is a well-established inflammatory marker, it does not capture the complexity of the inflammatory milieu, and additional markers such as IL-6, TNF-α, or interleukin-1β may offer a more nuanced understanding of the link between inflammation and muscle physiology [7,37]. Lastly, our assessment of dietary intake relied on a single 24 h recall, which may not reflect usual intake patterns, and residual confounding by unmeasured variables such as medication use (e.g., statins, corticosteroids), or hormonal status in women may have influenced the results.

Nevertheless, this study adds to the growing body of evidence that inflammation plays a meaningful role in muscle health. The use of a large, nationally representative dataset, stratified analysis by age and sex, and rigorous statistical adjustment enhance the robustness and generalizability of the findings. Future studies should prioritize prospective cohort designs and interventional trials to assess whether reducing hsCRP or targeting upstream inflammatory pathways can lead to meaningful improvements in muscle strength and function.

## 5. Conclusions

Our study demonstrates that elevated hsCRP levels are significantly associated with low muscle strength in middle-aged and older Korean adults, particularly among women. These results emphasize the role of systemic inflammation in the pathogenesis of muscle decline and suggest that hsCRP may serve as a practical biomarker for identifying individuals at increased risk of sarcopenia. Efforts to monitor and reduce chronic inflammation—through both clinical and lifestyle approaches—may represent a promising strategy for preserving muscle function and promoting healthy aging across the adult lifespan.

## Figures and Tables

**Table 1:** General characteristics of the subjects aged 19–39 years according to high sensitivity C-reactive protein.

Characteristics	Men			*p* Value *	Women			*p* Value *
	Normal (*n* = 1225)	Elevated (*n* = 364)	High (*n* = 131)		Normal (*n* = 1669)	Elevated (*n* = 338)	High (*n* = 169)	
Age, years	29.3 ± 0.2	30.3 ± 0.4	29.9 ± 0.6	0.011	29.5 ± 0.2	29.8 ± 0.4	30.4 ± 0.5	0.198
Household income				0.974				0.045
Low	98 (8.9)	27 (8.6)	13 (10.5)		105 (6.8)	27 (9.3)	14 (9.3)	
Middle-low	276 (22.6)	84 (23.0)	30 (21.0)		412 (25.1)	111 (30.7)	57 (34.2)	
Middle-high	420 (33.5)	133 (35.5)	40 (32.5)		582 (33.9)	106 (31.3)	54 (29.6)	
High	429 (35.0)	120 (32.9)	48 (35.9)		567 (34.2)	94 (28.8)	43 (26.9)	
Current alcohol consumer	935 (76.4)	263 (71.6)	95 (74.7)	0.236	1015 (63.4)	177 (55.0)	96 (52.8)	0.003
Current smoker	480 (38.6)	141 (37.3)	55 (43.0)	0.565	123 (8.4)	24 (6.2)	8 (5.6)	0.277
Regular resistance exercise	396 (33.4)	81 (22.2)	26 (23.3)	<0.001	268 (17.1)	34 (10.6)	23 (14.3)	0.029
Medical condition ^†^	9 (0.7)	6 (1.2)	3 (1.6)	0.383	18 (1.0)	5 (1.2)	8 (4.5)	0.001
Body mass index, kg/m^2^	23.9 ± 0.1	27.3 ± 0.3	27.5 ± 0.5	<0.001	21.4 ± 0.1	24.6 ± 0.3	26.7 ± 0.5	<0.001
hsCRP, mg/L	0.46 ± 0.01	1.63 ± 0.03	6.14 ± 0.32	<0.001	0.41 ± 0.01	1.65 ± 0.03	5.29 ± 0.20	<0.001
Maximal handgrip strength, Kg	42.7 ± 0.2	42.7 ± 0.5	41.5 ± 0.6	0.175	24.8 ± 0.1	25.0 ± 0.3	25.6 ± 0.4	0.180
Low muscle strength	26 (2.2)	9 (3.0)	4 (2.2)	0.693	102 (6.2)	24 (7.8)	12 (7.2)	0.605
Total energy intake, kcal/day	2549 ± 36	2510 ± 63	2419 ± 92	0.416	1783 ± 21	1780 ± 55	1805 ± 83	0.964
Dietary protein intake, g/day	97.4 ± 1.7	96.2 ± 3.0	93.3 ± 4.4	0.687	66.2 ± 1.0	64.5 ± 2.2	65.6 ± 3.6	0.775
Dietary vitamin C intake, mg/day	64.2 ± 2.3	64.3 ± 6.5	69.4 ± 9.7	0.874	55.9 ± 1.8	57.4 ± 4.4	58.9 ± 5.4	0.840
Dietary vitamin E intake, mg α-TE/day	8.23 ± 0.15	7.93 ± 0.28	7.88 ± 0.41	0.530	6.17 ± 0.12	6.22 ± 0.21	6.41 ± 0.59	0.915
Dietary β-carotene intake, μg/day	2627 ± 70	2503 ± 124	2558 ± 220	0.670	2161 ± 60	2594 ± 173	4045 ± 1996	0.038

Data are expressed as means ± SE for continuous variables or number (%) for categorical variables. * Differences were determined by ANOVA for continuous variables or Rao-Scott chi-square tests for categorical variables using SPSS version 26. ^†^ Any prevalence of type 2 diabetes, cancers, stroke, cardiovascular diseases, or osteoarthritis.

**Table 2:** General characteristics of the subjects aged 40–64 y according to high sensitivity C-reactive protein.

Characteristics	Men			*p* Value *	Women			*p* Value *
	Normal (*n* = 1225)	Elevated (*n* = 364)	High (*n* = 131)		Normal (*n* = 1669)	Elevated (*n* = 338)	High (*n* = 169)	
Age, years	50.8 ± 0.2	51.3 ± 0.3	51.7 ± 0.5	0.051	51.2 ± 0.2	52.5 ± 0.3	52.0 ± 0.5	<0.001
Household income				<0.001				0.009
Low	169 (7.9)	60 (7.5)	43 (16.1)		292 (9.1)	99 (13.3)	38 (14.7)	
Middle-low	395 (18.8)	159 (24.4)	65 (21.9)		685 (22.6)	177 (23.7)	54 (20.5)	
Middle-high	597 (31.0)	185 (29.1)	70 (27.5)		873 (30.2)	199 (30.6)	84 (33.4)	
High	800 (42.3)	230 (39.0)	82 (34.4)		1094 (38.1)	216 (31.4)	76 (31.4)	
Current alcohol consumer	1460 (74.9)	459 (73.9)	187 (71.4)	0.537	1296 (46.5)	253 (36.7)	105 (44.2)	<0.001
Current smoker	699 (35.8)	283 (46.7)	117 (44.1)	<0.001	126 (4.7)	39 (5.7)	19 (8.9)	0.055
Regular resistance exercise	519 (26.6)	148 (23.7)	51 (20.4)	0.102	506 (17.2)	80 (10.8)	33 (13.1)	0.001
Medical condition ^†^	327 (13.5)	100 (13.4)	49 (17.1)	0.346	532 (16.7)	156 (23.6)	57 (22.4)	<0.001
Body mass index, kg/m^2^	24.4 ± 0.1	25.5 ± 0.1	25.2 ± 0.3	<0.001	23.1 ± 0.1	25.3 ± 0.2	25.6 ± 0.3	<0.001
hsCRP, mg/L	0.50 ± 0.01	1.62 ± 0.02	6.30 ± 0.29	<0.001	0.46 ± 0.00	1.65 ± 0.02	5.60 ± 0.22	<0.001
Maximal handgrip strength, Kg	42.0 ± 0.2	41.6 ± 0.3	40.5 ± 0.5	0.018	24.6 ± 0.1	24.4 ± 0.2	24.1 ± 0.3	0.333
Low muscle strength	44 (1.9)	22 (2.4)	13 (4.3)	0.051	188 (6.3)	62 (9.3)	21 (6.5)	0.029
Total energy intake, kcal/day	2432 ± 24	2401 ± 47	2427 ± 74	0.833	1692 ± 15	1670 ± 27	1730 ± 48	0.533
Dietary protein intake, g/day	86.2 ± 1.0	84.6 ± 2.0	83.8 ± 3.2	0.604	60.9 ± 0.7	58.7 ± 1.2	63.5 ± 2.1	0.081
Dietary vitamin C intake, mg/day	69.2 ± 2.2	68.4 ± 4.1	64.1 ± 4.2	0.545	66.8 ± 1.6	65.8 ± 4.8	65.3 ± 4.8	0.940
Dietary vitamin E intake, mg α-TE/day	7.94 ± 0.14	7.63 ± 0.19	7.28 ± 0.30	0.078	5.98 ± 0.08	5.77 ± 0.13	6.60 ± 0.32	0.043
Dietary β-carotene intake, μg/day	3295 ± 74	3483 ± 147	3279 ± 192	0.517	2817 ± 58	2781 ± 124	2906 ± 177	0.850

Data are expressed as means ± SE for continuous variables or number (%) for categorical variables. * Differences were determined by ANOVA for continuous variables or Rao-Scott chi-square tests for categorical variables using SPSS version 26. ^†^ Any prevalence of type 2 diabetes, cancers, stroke, cardiovascular diseases, or osteoarthritis.

**Table 3:** General characteristics of the subjects aged ≥65 y according to high sensitivity C-reactive protein.

Characteristics	Men			*p* Value *	Women			*p* Value *
	Normal (*n* = 1225)	Elevated (*n* = 364)	High (*n* = 131)		Normal (*n* = 1669)	Elevated (*n* = 338)	High (*n* = 169)	
Age, years	72.3 ± 0.2	73.0 ± 0.3	73.2 ± 0.4	0.035	72.4 ± 0.1	73.0 ± 0.3	73.9 ± 0.5	0.003
Household income				0.094				0.125
Low	395 (36.0)	172 (42.0)	100 (49.0)		690 (47.5)	258 (52.7)	121 (57.5)	
Middle-low	319 (21.6)	108 (26.0)	56 (26.6)		352 (25.4)	130 (24.5)	36 (18.0)	
Middle-high	198 (19.7)	68 (18.1)	20 (13.9)		187 (14.8)	55 (13.1)	26 (16.7)	
High	135 (14.7)	48 (13.9)	20 (10.5)		139 (12.3)	43 (9.7)	11 (7.9)	
Current alcohol consumer	621 (59.3)	217 (56.5)	106 (56.8)	0.648	244 (19.4)	89 (17.3)	26 (11.3)	0.034
Current smoker	173 (16.4)	81 (20.4)	35 (19.0)	0.302	25 (2.2)	10 (2.1)	6 (2.3)	0.983
Regular resistance exercise	292 (27.9)	109 (30.9)	33 (17.0)	0.005	130 (10.5)	38 (9.5)	15 (8.4)	0.707
Medical condition ^†^	437 (40.5)	163 (38.7)	83 (37.3)	0.729	746 (54.0)	279 (55.4)	121 (59.7)	0.437
Body mass index, kg/m^2^	23.6 ± 0.1	24.1 ± 0.2	23.6 ± 0.2	0.042	24.2 ± 0.1	25.2 ± 0.2	24.9 ± 0.3	<0.001
hsCRP, mg/L	0.50 ± 0.01	1.64 ± 0.04	6.70 ± 0.37	<0.001	0.50 ± 0.01	1.65 ± 0.03	6.73 ± 0.42	<0.001
Maximal handgrip strength, Kg	33.4 ± 0.2	33.0 ± 0.4	30.8 ± 0.6	<0.001	20.0 ± 0.2	19.3 ± 0.3	18.3 ± 0.4	<0.001
Low muscle strength	229 (21.1)	91 (21.1)	67 (34.9)	<0.001	440 (31.3)	189 (38.1)	92 (45.3)	<0.001
Total energy intake, kcal/day	1925 ± 25	1951 ± 52	1948 ± 53	0.839	1471 ± 20	1493 ± 34	1397 ± 58	0.343
Dietary protein intake, g/day	65.3 ± 1.0	64.7 ± 2.0	65.4 ± 2.6	0.960	47.7 ± 0.7	48.4 ± 1.3	44.7 ± 2.1	0.295
Dietary vitamin C intake, mg/day	60.0 ± 2.3	58.5 ± 4.7	55.9 ± 4.5	0.707	53.1 ± 2.1	50.9 ± 2.9	62.8 ± 10.1	0.496
Dietary vitamin E intake, mg α-TE/day	5.87 ± 0.12	5.93 ± 0.54	5.55 ± 0.25	0.499	4.55 ± 0.10	4.37 ± 0.14	4.07 ± 0.27	0.170
Dietary β-carotene intake, μg/day	2960 ± 102	2968 ± 198	2529 ± 159	0.067	2364 ± 77	2261 ± 137	2087 ± 181	0.342

Data are expressed as means ± SE for continuous variables or number (%) for categorical variables. * Differences were determined by ANOVA for continuous variables or Rao-Scott chi-square tests for categorical variables using SPSS version 26. ^†^ Any prevalence of type 2 diabetes, cancers, stroke, cardiovascular diseases, or osteoarthritis.

**Table 4:** Association between high sensitivity C-reactive protein level and maximal handgrip strength of Korean adults by age and sex.

	Unadjusted		Model 1 *		Model 2 †		Model 3 ‡	
	β (SE)	*p*-Value	β (SE)	*p*-Value	β (SE)	*p*-Value	β (SE)	*p*-Value
19–39 years								
Men	−0.144 (0.088)	0.103	−0.327 (0.085)	<0.001	−0.353 (0.080)	<0.001	−0.350 (0.081)	<0.001
Women	0.138 (0.074)	0.064	−0.141 (0.080)	0.078	−0.129 (0.081)	0.112	−0.130 (0.081)	0.110
40–64 years								
Men	−0.169 (0.069)	0.014	−0.193 (0.067)	0.004	−0.136 (0.066)	0.041	−0.136 (0.066)	0.041
Women	−0.054 (0.047)	0.251	−0.104 (0.049)	0.035	−0.095 (0.048)	0.050	−0.096 (0.048)	0.048
≥65 years								
Men	−0.212 (0.092)	0.022	−0.175 (0.097)	0.071	−0.137 (0.095)	0.148	−0.138 (0.096)	0.151
Women	−0.164 (0.060)	0.006	−0.123 (0.051)	0.017	−0.160 (0.034)	<0.001	−0.160 (0.051)	0.002

* Model 1: adjusted for age, body mass index, and total energy intake. † Model 2: model 1 plus household income, alcohol consumption, smoking, resistance exercise, and medical condition. ‡ Model 3: model 2 plus energy from protein.

**Table 5:** Risks of low muscle strength according to levels of high sensitivity C-reactive protein among Korean adults by age and sex.

hsCRP Level	Unadjusted		Model 1 *		Model 2 †		Model 3 ‡	
	OR	95% CI	OR	95% CI	OR	95% CI	OR	95% CI
19–39 years								
Men								
Normal	1 (ref)		1 (ref)		1 (ref)		1 (ref)	
Elevated	1.37	0.62, 3.05	1.96	0.82, 4.69	1.48	0.59, 3.71	1.50	0.59, 3.79
High	1.00	0.30, 3.32	1.36	0.40, 4.60	1.16	0.32, 4.16	1.20	0.33, 4.32
Women								
Normal	1 (ref)		1 (ref)		1 (ref)		1 (ref)	
Elevated	1.26	0.78, 2.04	1.50	0.92, 2.44	1.32	0.80, 2.18	1.31	0.79, 2.18
High	1.16	0.60, 2.28	1.55	0.76, 3.15	1.50	0.74, 3.05	1.49	0.74, 3.02
40–64 years								
Men								
Normal	1 (ref)		1 (ref)		1 (ref)		1 (ref)	
Elevated	1.25	0.68, 2.32	1.38	0.74, 2.57	1.30	0.67, 2.50	1.30	0.67, 2.49
High	2.31	1.15, 4.66	2.25	1.10, 4.63	1.81	0.88, 3.73	1.82	0.88, 3.74
Women								
Normal	1 (ref)		1 (ref)		1 (ref)		1 (ref)	
Elevated	1.52	1.10, 2.10	1.56	1.11, 2.20	1.47	1.04, 2.09	1.47	1.04, 2.09
High	1.03	0.61, 1.73	1.08	0.63, 1.87	1.03	0.59, 1.78	1.03	0.59, 1.78
≥65 years								
Men								
Normal	1 (ref)		1 (ref)		1 (ref)		1 (ref)	
Elevated	1.00	0.73, 1.38	0.96	0.68, 1.36	0.97	0.68, 1.39	0.97	0.68, 1.39
High	2.00	1.38, 2.91	2.02	1.30, 3.15	1.71	1.06, 2.75	1.71	1.06, 2.75
Women								
Normal	1 (ref)		1 (ref)		1 (ref)		1 (ref)	
Elevated	1.35	1.06, 1.73	1.33	1.03, 1.73	1.34	1.04, 1.73	1.34	1.04, 1.74
High	1.82	1.29, 2.57	1.57	1.08, 2.28	1.66	1.15, 2.41	1.66	1.14, 2.42

* Model 1: adjusted for age, body mass index, and total energy intake. † Model 2: model 1 plus household income, alcohol consumption, smoking, resistance exercise, and medical condition. ‡ Model 3: model 2 plus energy from protein.

## Data Availability

The data (KNHANES VII) presented in this study are available at https://knhanes.kdca.go.kr/knhanes/main.do (accessed on 17 August 2025).
